# Researchers on research integrity: a survey of European and American researchers

**DOI:** 10.12688/f1000research.128733.1

**Published:** 2023-02-16

**Authors:** Nick Allum, Abigail Reid, Miriam Bidoglia, George Gaskell, Noémie Aubert-Bonn, Ivan Buljan, Simon Fuglsang, Serge Horbach, Panagiotis Kavouras, Ana Marušić, Niels Mejlgaard, Daniel Pizzolato, Rea Roje, Joeri Tijdink, Giuseppe Veltri

**Affiliations:** 1 Department of Sociology, University of Essex, Colchester, UK; 2 Department of Methodology, London School of Economics and Political Science, London, UK; 3 Department of Ethics, Law and Humanities, VU University Amsterdam, Amsterdam, The Netherlands; 4 Department of Research in Biomedicine and Health, University of Split, Split, Croatia; 5 Department of Political Science, The Danish Centre for Studies in Research and Research Policy, Aarhus University, Aarhus, Denmark; 6 Department of Materials Science and Engineering, School of Chemical Engineering, National Technical University of Athens, Athens, Greece; 7 Centre for Bioethics and Law, Catholic University of Leuven, Leuven, Belgium; 8 Department of Philosophy, Vrije Universiteit Amsterdam, Amsterdam, The Netherlands; 9 Department of Sociology and Social Research, University of Trento, Trento, Italy

**Keywords:** research integrity, meta-research, survey, questionable research practices

## Abstract

Background: Reports of questionable or detrimental research practices (QRPs) call into question the reliability of scientific evidence and the trustworthiness of research. A critical component of the research ecosystem is the organization within which research takes place. We conducted a survey to explore the attitudes and beliefs of European and American researchers about the organisations in which they work, their own research practices and their attitudes towards research integrity and research integrity policies.

Methods: We administered an online survey (International Research Integrity Survey (IRIS)) to 2,300 active researchers based in the US and 45,000 in Europe (including UK, Norway, Iceland and Switzerland).  We employed a stratified probability sample of the authors of research articles published between 2016 and 2020 included in Clarivate’s Web of Science citation database. Coverage includes researchers in the humanities, social sciences, natural sciences and medical sciences, who hold at least a master’s level degree.

Results: In comparison to researchers in the US, European researchers admit to more QRPs and are less confident in maintaining high research integrity (RI) standards. In the US and Europe, many researchers judge their organization to fall short of best RI practice. All researchers recognize the benefits of RI, reliable knowledge and the trust of colleagues and the public, and there is support for RI training particularly among Europeans.

Conclusion: To create and maintain a culture of integrity in scientific research, a collective commitment from researchers, their institutions and funders is needed. Researchers rely on many channels of communication about research integrity and thus the involvement of many different participants in the research system is required to make improvements. Policies must be developed to reinforce best practice rather than being seen as an irrelevance to the real business of research.

## Introduction

Scientific research evolves within a set of processes and cultures that potentially shape the integrity/reproducibility of results. Without good research practices, the credibility and trustworthiness of research is called into doubt. Part of this research ecosystem is the organization within which research takes place. We conducted a survey to explore the attitudes and beliefs of 47,000 European and American researchers about the organisations in which they work and their attitudes and beliefs about research integrity and research integrity policies.

In 1992, reports of falsification, plagiarism and misconduct in science led the US National Academies of the Sciences, Engineering and Medicine (NASEM) to publish ‘Responsible Science: Ensuring the Integrity of the Research Process’ ( National Academy of Sciences (US), National Academy of Engineering (US) and Institute of Medicine (US) Panel on Scientific Responsibility and the Conduct of Research, 1992). Three decades later, in response to serious cases of scientific misconduct, a disturbing increase in retractions, and low rates of reproducibility, NASEM published a new report in 2017, ‘Fostering Integrity in Research’ ( National Academies of Sciences and Medicine, 2017). The National Science Foundation (NSF), which funds 27 % of federally supported research in universities and colleges in the US ( At a Glance|NSF - National Science Foundation, n.d.), not only sets out detailed protocols on research integrity (RI) but also requires institutions submitting a proposal to certify that they provide training and oversight in the ethical conduct of research to all those supported by NSF. In addition to NSF’s oversight, the Office of Inspector General and the Office of Research Integrity have powers to investigate research misconduct and receive reports of possible misconduct or fraud from whistle-blowers ( Whistleblower Protection|Office of Inspector General, n.d.).

The European Science Foundation and the All European Academies (ALLEA) took up the cause of RI in the early 2000s. Unlike the federal system of the US, Europe comprises independent and heterogenous countries, with autonomous political and educational systems and varying research cultures ( Godecharle *et al*., 2013, 2014). Nonetheless, the recently updated European Code of Conduct for Research Integrity which defines four fundamental principles of RI: reliability, honesty, respect and accountability ( The European Code of Conduct for Research Integrity - ALLEA, n.d.), calls for consistency across the European Member States in the handling of violations, describing the professional, legal and ethical responsibilities in a framework for self-regulation. Last year saw the introduction of an important pan-European policy; the European Union’s €95.5 billion research fund for 2021-2027 stipulates that to be awarded research funding, applicants must confirm compliance with the ALLEA Code of Conduct and have appropriate procedures, policies and structures in place to foster responsible research practices, to prevent questionable research practices and research misconduct, and to handle allegations of breaches of the principles and standards in the Code of Conduct ( European Commission, 2022).

Within this context of external RI codes, expectations and requirements, we investigate the state of RI, as viewed by active researchers in the US and Europe. Europe and the US have taken different approaches to the promotion of research integrity, raising the question ‘what works?’ What should funding bodies, organisations producing research and researchers themselves be doing? We contribute evidence useful in answering these questions by illuminating the researchers’ perspectives, using data from a large-scale international survey of active researchers in the medical, natural, social sciences and humanities.

In it, we asked researchers about their attitudes to, and awareness of, RI policies in their organisations. We asked how much support they needed and in which areas. We also asked about researchers’ confidence in their ability to carry out high quality research and asked about their engagement in questionable research practices (QRPs). We focus particularly on comparisons between researchers in Europe and within the US, but also explore differences by scientific field, sex, career stage, employment contract and industry sector.

## Methods

### Ethics

Ethical approval for conducting the survey was obtained from the University of Essex, UK, Faculty of Social Sciences Ethics Committee (ETH2021-0441). The approval document can also be found on OSF (DOI 10.17605/OSF.IO/XB9RK). Informed consent was obtained by the provision of information to participants before they agreed to respond by clicking the survey link.

### International Research Integrity Survey (IRIS)

The survey was conducted as part of a larger study on RI. Standard Operating Procedures for Research Integrity (SOPs4RI) is a four-year project funded by the European Union under the ‘Science with and for Society’ programme ( SOPs4RI – Promoting excellent research, n.d.). IRIS is based on a systematic, stratified probability sample of the authors of research articles published between 2016 and 2020 included in Clarivate’s Web of Science citation database. The full sample from 34 countries is over 60,000 respondents. The focus of this paper is 2300 researchers based in the US and 45,000 in Europe (including UK, Norway, Iceland and Switzerland). Few, if any, previous surveys on RI and related issues have been based on probability designs or cover such a wide range of research fields. Since 2004, a number of surveys have asked researchers about RI ( Titus *et al*., 2008; Van den Eynden *et al*., 2016; Woolston, 2020), most recently a national study in the Netherlands ( Gopalakrishna *et al*., 2022) but IRIS is the first survey to feature such a broad, representative international sample.

The online questionnaire focuses on the behaviors of researchers but also on what they think about RI practices and policies in their organizations. Respondents were asked about their self-confidence in supporting research with integrity; if they consider their organization is effective in delivering best RI practices; how much institutional oversight of RI is desirable; whether they had engaged in selected questionable (or detrimental) research practices (QRPs); how closely their organization aligns with best practice; what are the dominant channels of communication on RI topics, what would motivate them to engage with RI policies and procedures and on which topics they would welcome additional support.

### The sample

The population of interest for this study was active researchers in the humanities, social sciences, natural sciences (including technical science), and medical sciences (including bio-medicine), who hold at least a master’s level degree and produce research for commercial or academic institutions within the EU, EFTA, U.K. and the US. The way we divide researchers into these broad fields follows the OECD Frascati Manual. We show a detailed breakdown of how subfields are allocated within each of the four main fields, along with their frequency distributions in the *Extended data* ( Allum *et al*., 2022). The sampling frame was the Clarivate Web of Science bibliographic database, which contains details of publications produced by researchers in 21,894 scientific journals, books and conference proceedings. The sample was constructed from a background population of academics, identified in the bibliographic database, Web of Science (WoS). WoS contains article metadata for more than a million research articles annually. From these records we extracted information on author names, affiliations and e-mail addresses, for all articles published in the period 2016-2020, where at least one author had an affiliation to an institution in one of the target countries. We downloaded 8,159,772 metadata records and retrieved 3,929,283 e-mail addresses, from which we were able to create 3,759,814 author profiles. Of these 3,072,372 were from our countries of interest.

Our objective was to obtain a sample that was both representative of the WoS population and contained sufficient numbers of observations within all countries and fields to enable robust comparisons to be made. To accomplish this, we generated a systematic sample with unequal selection probabilities with explicit and implicit stratification. We aimed to increase the precision of comparisons across four scientific fields by each country combinations through aiming for a similar effective sample size within each such combination. This naturally led to an unequal selection probability sample design with lower selection probabilities in those field-country combinations that have a larger number of publications in WoS. The explicit stratification categories were fully-crossed country by scientific field (natural, medical, social sciences and humanities) combinations. Within each such stratum a systematic sample was drawn, additionally using implicit stratification by a more granular indicator of scientific field and an indicator of the number of papers published by each author. The exceptions to this procedure include those countries, or fields within some countries, where the total number of authors was smaller than that required to achieve the planned effective sample size. In such situations all authors were included in the issued sample.

### The survey

The survey was conducted entirely online, in English, using the Qualtrics platform. The survey rationale was developed and agreed in consultation with project partners as detailed in the survey protocol document ( Reid and Allum, 2021). Survey questions were developed between November 2020 and April 2021 by authors NA and AR, guided by topic experts within the group of project partners. The full survey included sections covering: structural or demographic variables; values, beliefs and attitudes in relation to science practices, research integrity policies and the role of organisations in implementing them; the current research integrity landscape, including awareness of and satisfaction with current research integrity arrangements; personal efficacy and behaviour; and receptivity towards research integrity policies including specific examples of standard operating procedures. A set of items asking about questionable research practices (QRPs) was drawn from past research on the following criteria: breadth of coverage across research integrity areas, applicable regardless of discipline and ranging from common to rare behaviors ( Gopalakrishna *et al*., 2022; Schneider *et al*., In preparation).

Cognitive testing of the questionnaire was conducted via online meetings due to COVID-19 during February and March 2021. A simple random sample of 5000 people were invited to take part in pilot testing from April to May 2021, following which some changes were made. Invitations and reminders to take part in the survey were distributed from 22 ^nd^ June – 28 ^th^ July 2021 and the survey closed on 14 ^th^ September 2021.

The invitation emails included information about the project and funder, with links to the Qualtrics survey and to instructions for how to opt out from further communication. In addition, it included information about how the individual had been selected, the scope and purpose of the research for which personal data about them would be collected, how their personal data would be used, who would have access to it, the benefits of participation, and their right to withdraw at any time, including instructions on how to do so. Respondents were told that starting the survey after reading the information supplied implied written consent to participate. The full text of the emails and questionnaire can be found in the *Extended data* ( Allum *et al*., 2022).

73,757 people responded to the survey. Of these, 1,602 were ineligible due to their country of employment being outside our target countries, which were EU, UK, Norway, Switzerland, USA and Canada. A further 6,391 were excluded as they completed less than 25 % of the survey, which gave no information beyond demographics. Lastly, those who did not state that they were trained to at least master’s level were removed. The remaining 64,074 cases were retained. The overall response rate, computed using the American Association for Public Opinion Research’s standard definitions, was 7.2% (AAPOR Response Rate 2) ( American Association for Public Opinion Research, 2016). For this study, we selected only those who had completed 75% of the survey or more and who were employed in Europe or the United States of America at the time of completing the survey.

### Weighting

We computed weights that we apply in our analyses to correct for the unequal selection probabilities of cases inherent in the sample design and for biases caused by differential non-response. Not all the authors in WoS had the same initial probability of selection, depending on the sizes of the WoS sub-populations used in the stratified design. We aimed to gather approximately 500 responses in each scientific field in each country. Hence those authors in smaller countries that had few authors in WoS had a higher probability of selection than those in countries that had much greater representation. The weighting reflects these relative selection probabilities. In addition to design-related adjustments, we used the information about the WoS authors that we included in the sample design to estimate the overall probability of responding, adjusting for both the study design and non-response. We modelled this using logistic regression. A binary variable that indicated whether a sample member provided a usable response to the survey (i.e. answered more than 25% of the questions) was specified as the dependent variable. The independent variables were country, field, country x field, number of papers and granular subfield. The model therefore takes into account simultaneously the unequal selection probabilities and the differential non-response propensity. The weight variable we derive from estimating this model this was computed as the inverse of the predicted response probability for each respondent and normalised so that the final weighted sample size matched the unweighted sample size. We also trimmed this weight at the 99 ^th^ percentile so as not to over-inflate the design effect.

### Analysis/statistical methods

Our analysis is largely descriptive and was not preregistered. We present mainly two-way cross-tabulations and bar charts for outcome variables of interest, split by region, scientific field, sex, employment contract, industrial sector and career stage. In some sections, we also present OLS multiple regression analyses to adjust for potential confounding relationships. In some cases, we show 95% confidence intervals around estimates. In many cases we do not, as tables would be unwieldy, but we include unweighted sample sizes. For all data tables, with confidence intervals for all of our estimates, see *Extended data* ( Allum *et al*., 2022). Standard errors were estimated using Taylor linearization. All analyses were carried out using Stata 17 software with the svy prefix command to adjust for the sample design and weighting. Tables showing all of the weighted and unweighted frequencies for all variables are included in the *Extended data* ( Allum *et al*., 2022). Details oƒ recodes and derived variables are described in the relevant sections alongside the results. All oƒ the Stata code used for preparation and analysis included in the *Extended data* along with the full dataset, permitting all of our results to be reproduced independently.

## Results

### Confidence in integrity of own research

We asked respondents how confident they are that they are currently meeting high RI standards. While more than 95% of researchers report at least ‘some’ confidence, 74% of researchers in the US say they are ‘very confident’, compared to just 52% of European researchers (see Table 1).

**Table 1.** How confident that research is meeting high integrity standards.

n=47,512	Overall	US	Europe
	%	%	%
Very confident	59	74	52
Somewhat confident	38	24	43
Not very confident	3	2	4
Not at all confident	0	0	0

This transatlantic confidence gap persists across researchers of different career stages, employment contracts and scientific fields (see Table 2). More established researchers, both in length of career and security of contract, report greater levels of confidence in the integrity of their research, across Europe and in the US.

**Table 2.** How confident that research is meeting high integrity standards by field, sector, contract, stage and sex.

		Very confident		Somewhat confident		Not very confident		Not at all confident	
		US	Eur	US	Eur	US	Eur	US	Eur
Field n=45,709	Natural	75	53	23	42	1	4	0	0
	Medical	76	51	22	44	1	4	1	0
	Social	67	50	30	46	1	3	1	0
	Humanities	81	55	17	41	2	3	0	0
Sector n=47,464	Academia	74	53	24	42	2	4	0	0
	Other	74	49	24	47	1	4	0	0
Contract n=44,544	Permanent	76	54	22	43	2	4	0	0
	Temporary	66	48	33	46	1	5	0	0
Stage n=44,844	Early	64	43	34	50	1	6	0	1
	Mid	69	52	28	44	1	4	1	0
	Later	82	62	16	35	2	2	0	0
Sex n=46,836	Female	69	49	29	46	1	4	1	0
	Male	78	54	20	42	2	4	0	0

In the US, high confidence in meeting RI standards rises from 64% among early career researchers to 82% in later career. The comparative figures for Europe are 43% (early career) and 62% (later career). In the US, those with permanent positions register 76% compared to 66% for those with temporary positions. The respective figures in Europe are far lower; 54% and 48%.

The same pattern emerges across the disciplinary fields. A comparison between the US and Europe in the natural, medical, social sciences and humanities shows approximately 20% fewer reporting high confidence in Europe across all fields.

## Confidence in organization to ensure integrity

We asked researchers how confident they are in their own organization’s effectiveness in ensuring that appropriate standards of research are maintained (the five response alternatives ranged from ‘complete’ to ‘no confidence’). In the US, 42% of researchers have either ‘complete’ or a ‘great deal’ of confidence in their organization’s effectiveness, as do 34% in Europe. At the same time, 22% of Americans and 29% of Europeans have ‘not much’ or ‘no’ confidence in their organization’s effectiveness in this regard (see Table 3).

**Table 3.** How much confidence that management is effective in ensuring high level of research integrity.

n=44863	Overall	US	Europe
	%	%	%
Complete confidence	7	9	6
A great deal of confidence	30	33	28
Some confidence	37	36	37
Not much confidence	20	17	22
No confidence	6	5	7

One indicator of the effectiveness of an organization’s RI policy is the extent of awareness by members of the organization. 63% of researchers in the US report that their organization has a policy on research integrity, while 32% say they don’t know. In Europe the figures are 47% and 41% respectively (see Table 4).

**Table 4.** Does research institution have a written statement on research integrity.

n=47806	Overall	US	Europe
	%	%	%
Yes	51	63	47
No	10	5	13
I don't know	38	32	41

Overall, it appears that researchers express more confidence in themselves than in their organizations and that there are non-trivial levels of ignorance about organizational RI statements. To what extent this is troubling depends in part on where the locus of responsibility for RI is seen to lie. It was found that 61% of respondents think that responsibility for high standards of research should be shared between the individual and the organization. However, 31% think that there should be no organizational oversight at all. These proportions vary little between US and European researchers (see Table 5).

**Table 5.** Responsibility for research integrity.

n=47625	Overall	US	Europe
*It is up to me to carry out research to the highest standard:*	%	%	%
Without any oversight from my organisation	31	29	32
With some oversight from my organisation	61	65	60
With a lot of oversight from my organisation	7	7	8

Finally, when we look at the relationship between self-confidence and confidence in the organization, we found that 62% of European researchers who are least confident in their own ability also have no confidence in their organization’s effectiveness. The corresponding figure for the US is only 36% (see Table 6). This suggests that there is room for greater and more effective organizational support in RI to mitigate existing concerns among some researchers.

**Table 6.** Confidence in organization (rows) by self-confidence (columns) to ensure high level of research integrity in Europe and the US.

n=47303	Very confident		Somewhat confident		Not very confident		Not at all confident	
	US	Europe	US	Europe	US	Europe	US	Europe
	%	%	%	%	%	%	%	%
Complete confidence	11	9	3	3	0	1	0	1
A great deal of confidence	36	34	28	24	9	7	0	4
Some confidence	34	33	46	43	23	31	33	8
Not much confidence	15	18	19	25	54	48	31	25
No confidence	5	6	3	6	15	13	36	62

### Questionable research practices

**Figure 1.**
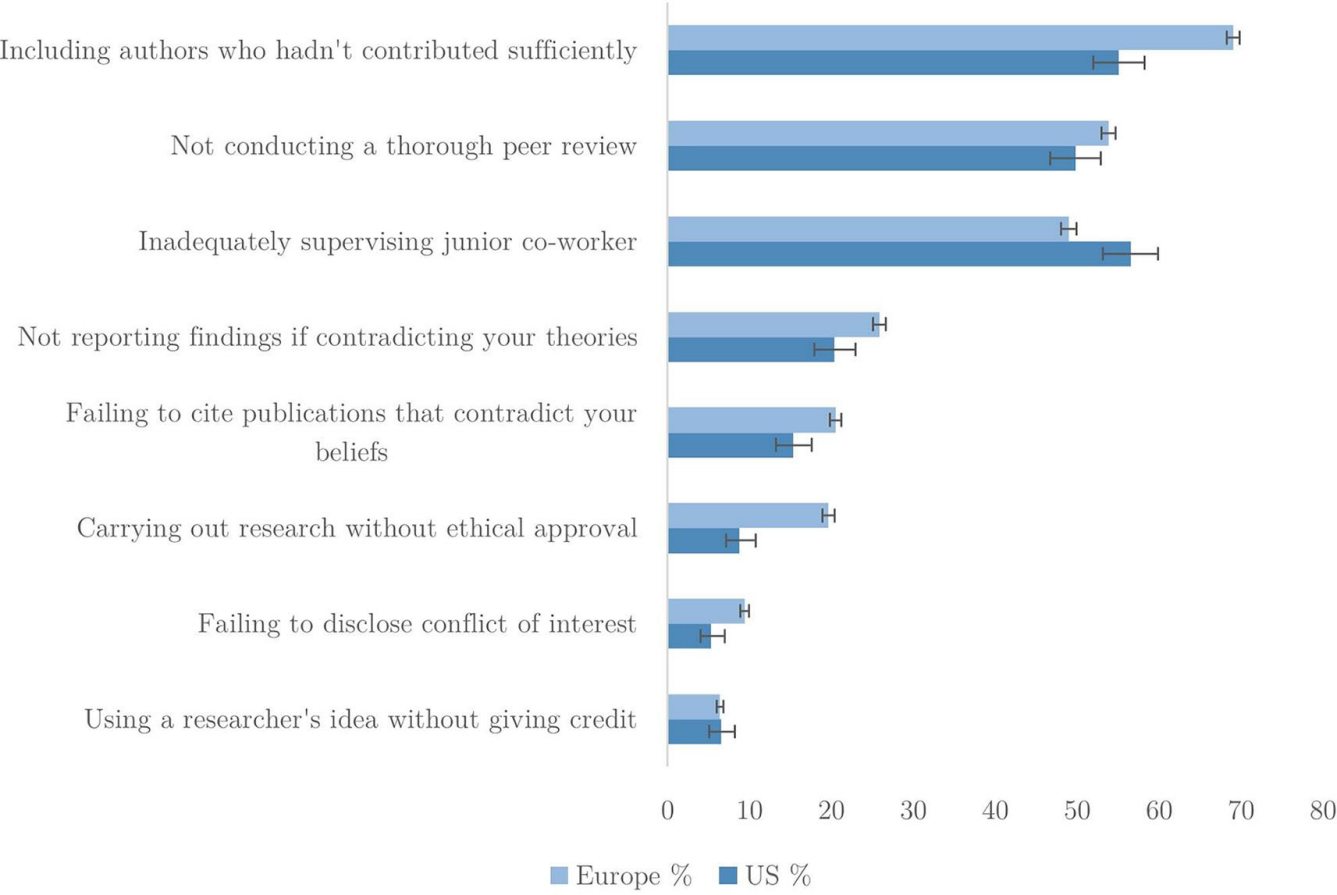
Percentage of researchers admitting QRPs have occurred in last 3 years.

Questionable research practices (QRPs) (often referred to as ‘detrimental research practices’) are a recognized challenge to RI (National Academies of Sciences and Medicine, 2017). They fall short of outright misconduct but represent transgressions of best practice, improper use of data and ethically questionable behavior. QRPs potentially diminish the quality and trustworthiness of scientific findings. We selected eight QRPs, based mainly on work by Schneider *et al.* (In preparation). Figure 1 shows short descriptions of the QRPs and the full wordings are presented in *Extended data* ( Allum *et al.,* 2022).

To introduce the issue of QRPs, respondents were presented with the following statement:

“The next few questions are about questionable research practices (QRPs). These are less than ideal research practices which might happen unintentionally. They are not research misconduct (i.e., fabrication, falsification, or plagiarism). We will present you with a set of research practices and ask you to what extent you have engaged in them when working towards producing your publications over the last three years.

Thinking about research carried out for your publications over the last three years, how often has the following occurred?”

The response alternatives were: often, sometimes, rarely, never, does not apply in my case.

Figure 1 shows the admitted occurrence of each applicable QRP in the US and Europe (error bars represent 95% confidence intervals). ‘Rarely’, ‘sometimes’ or ‘often’ are counted as an admission. For each item respondents could select ‘does not apply’, which excludes them from the results for the corresponding QRP.

The most common QRPs are *including authors who hadn’t contributed*, *not conducting a thorough review* and *inadequate supervision*, to which at least half of respondents admitted. Occurrence of remaining QRPs is acknowledged by between 10 and 20% of researchers. It is worth noting that the most frequent QRP – *inappropriate authorship* – does not necessarily implicate the respondent directly. For some, an affirmative response could mean that other investigator(s) may have been responsible. Hence, we might expect to see this QRP reported more often as it could include both individual and third-party decisions.

Turning to the contrast between US and European researchers, we find a consistent difference, with the latter admitting more QRPs on average than their US counterparts. The only exception to this is *inadequate supervision*, which US researchers are more likely to acknowledge. The largest disparities between regions are found for *carrying out research without ethical approval* and *failing to disclose conflicts of interest.* In both cases, the rate of admission is twice as high for European researchers.

**Figure 2.**
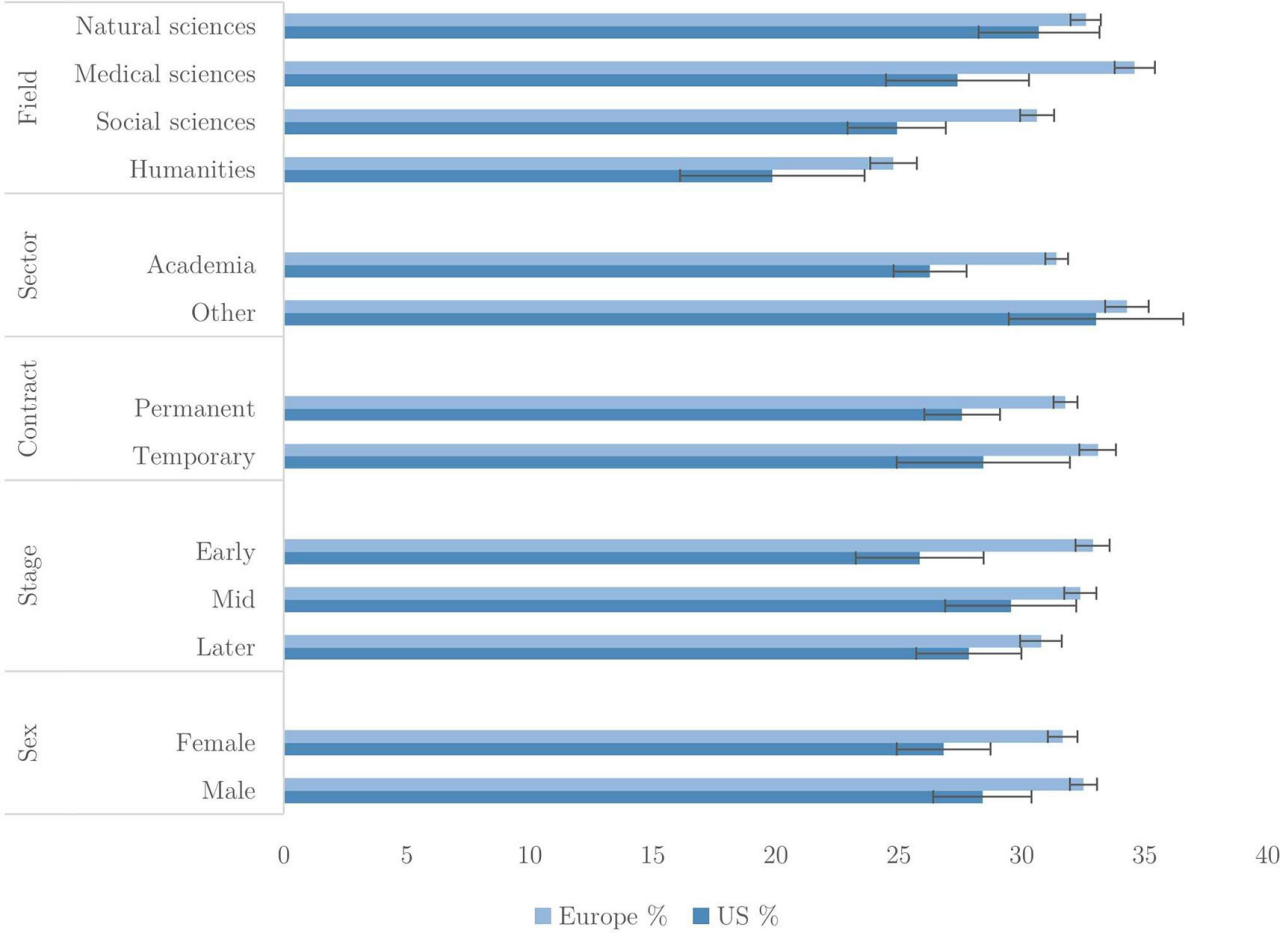
Percentage of applicable QRPs admitted to in last 3 years (n=40,317).

In Figure 2, we present the mean number of QRPs admitted out of the total applying to each researcher, expressed as percentages. For instance, a researcher who admitted three QRPs, and said that two out of the eight did not apply to them, would be assigned a score of 50% (i.e., three admitted out of six applicable). We show these for the US and Europe, with breakdowns by field, sector, employment contract, career stage and sex. Only one QRP – *carrying out research without ethical approval* – is indicated as not applicable by more than 20% of respondents, and the non-applicable percentage for most QRPs is in single digits (see *Extended data* for further detail).

The percentage of QRPs reported by Europeans exceeds that of US researchers, although in some cases the difference is within sampling error. Overall, researchers admit to between 20 to 35% of applicable QRPs. The largest differences are between fields. For example, European medical researchers admit to just under 35% of QRPs while humanities researchers admit to less than 25%. Natural and medical sciences display the greatest tendency for questionable practice, while social sciences and humanities researchers in both US and Europe show a lower self-reported prevalence. University researchers admit fewer QRPs than those in non-academic sectors.

**Figure 3.**
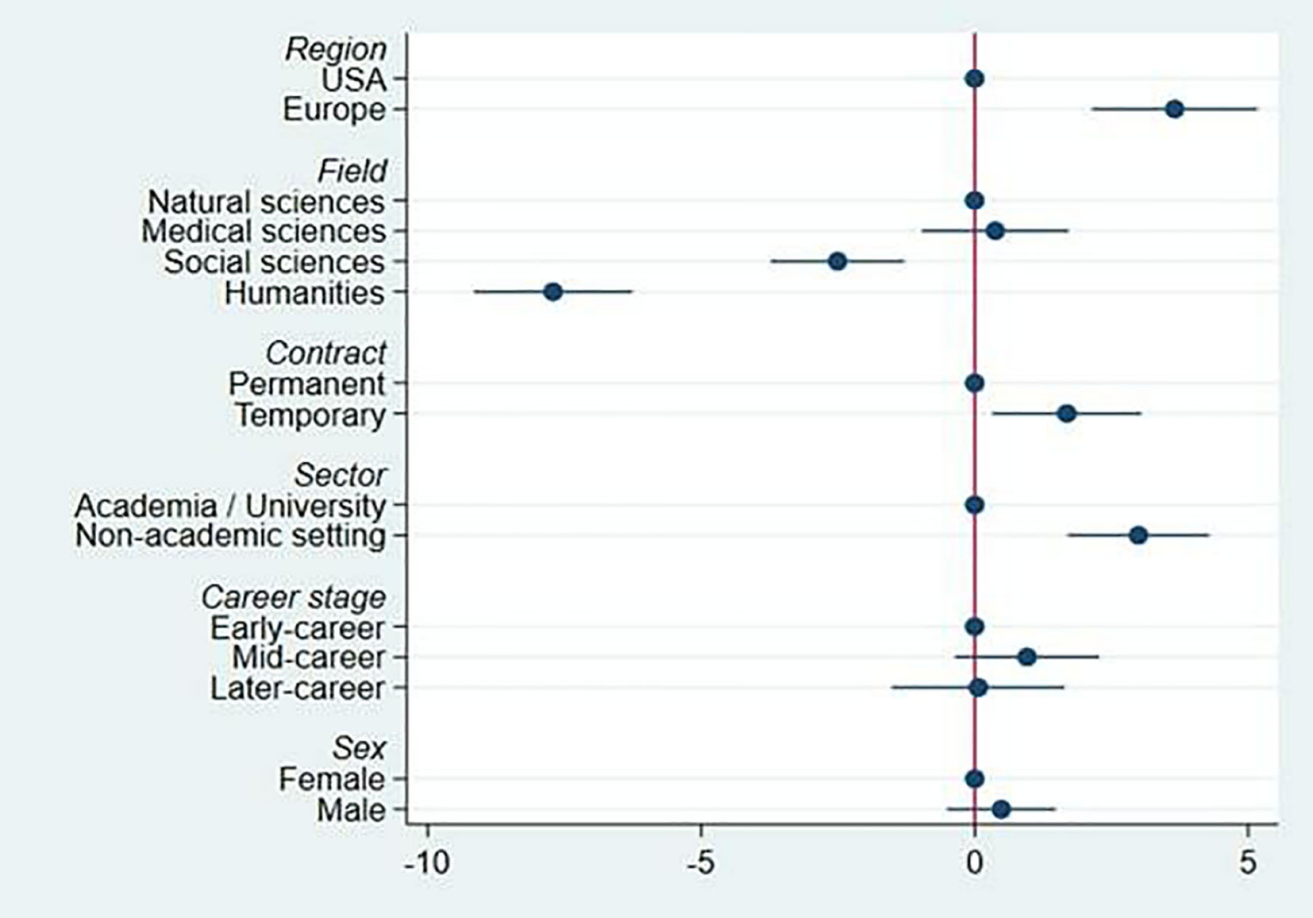
Regression of mean number of QRPs admitted, on country group, field, contract, sector, career stage and sex.

An interesting disparity emerges in relation to career stage; in Europe, early career researchers admit more QRPs than researchers in mid or late career, although the differences are not large. In the US, the pattern is reversed; they are reporting fewer QRPs than their more senior colleagues. Figure 3 presents results from an OLS multiple regression indicating the partial associations of the explanatory variables in Figure 2 with QRPs. Region, contract type, sector and field all show robust differences, after adjusting for all other factors.

### Perceptions of RI in organizations

What are the most important topics in organizational policies to support RI? In a series of focus groups and co-creation workshops with researchers from a wide variety of backgrounds, the SOPs4RI study found broad consensus on what these topics are and what counts as good practice (for full descriptions of these features, as presented to respondents, see *Extended data*) ( Mejlgaard *et al*., 2020; Sørensen *et al*., 2021). IRIS respondents were asked the extent to which their organization aligns with these policies and practices.

In a fully functioning research system, it might be expected that a high percentage of researchers would say their organization closely or very closely resembles best practice. In both US and Europe, researchers perceive a significant gap between best practice and their organization’s arrangements. In Table 7, we present the percentage of respondents who think that their organization ‘closely’ or ‘very closely’ resembles the ideal on the nine features of RI, broken down by field. Looking first at the bottom row of Table 7, the mean assessments across the nine RI features show that only 52% of researchers in the US and 45% in Europe consider their organizations to be close to the ideal.

**Table 7.** Perceptions of organizational RI practices in US and Europe.

	Overall (n=43,626)		Medical (n=6,601)		Social (n=12,760)		Natural (n=17,896)		Humanities (n=6,369)	
*Closely or very closely %*	US	Eur	US	Eur	US	Eur	US	Eur	US	Eur
Declaration of Interest	66	47	79	61	65	44	63	45	51	39
Working Environment	61	54	75	60	61	55	56	53	45	48
Data Management	60	54	72	54	58	49	57	56	56	50
Research Collaboration	52	44	63	47	49	40	51	45	34	38
Ethics Structures	51	42	73	67	55	45	40	33	38	34
Publication & Comms	49	54	55	55	42	49	52	55	33	48
Integrity Breaches	48	31	64	40	47	32	43	28	35	26
Supervision & Mentoring	44	42	57	44	38	40	44	42	28	37
Integrity Training	38	20	60	29	36	21	32	18	14	17
***Mean assessment***	***52***	***45***	***67***	***51***	***50***	***42***	***48***	***42***	***38***	***38***

* RI features ordered by mean assessment, closest to least close resemblance (US); logit models for each RI area showing the statistical significance of field and region differences can be found in SM table 11.

Considering transatlantic disparities within each field, only in the case of humanities is there no mean overall difference, although this masks variation within each of the elements. Only 38% in the US and 21% in Europe think *integrity training* is close to the ideal. Here then, is an area where organizations have considerable scope to improve the situation. Only a minority of researchers in both the US and Europe regard organizational practice around *supervision, integrity breaches* and *publications and communications* to be close to ideal. In all but the latter topic, US organizations are viewed as more closely matching the ideal than those in Europe.

### Influencers

We have shown that the amount of support provided by researchers’ organizations is regarded by many as less than ideal. At the same time, some researchers do not think that the responsibility for RI lies with their organization at all. This raises the question of who are the actors, communities and organizations whose opinions are most valued? For the many researchers, it is their scholarly community – those that publish in the same journals and attend the same conferences – whose opinions researchers value the most. This is true for 76% of Americans and 62% of Europeans. Researchers’ departments, organizations and professional bodies are each cited by no more than 12% of respondents as having the most important opinions (see Table 8).

**Table 8.** Whose opinion about your research do you value the most?

n=47769	Overall	US	Europe
	%	%	%
My department's or centre's	12	8	14
My organization's	6	4	7
Researchers in the country I am currently working	7	5	7
Professional societies I am affiliated with	9	6	10
My scholarly community	66	76	62

### Communication channels

**Figure 4.**
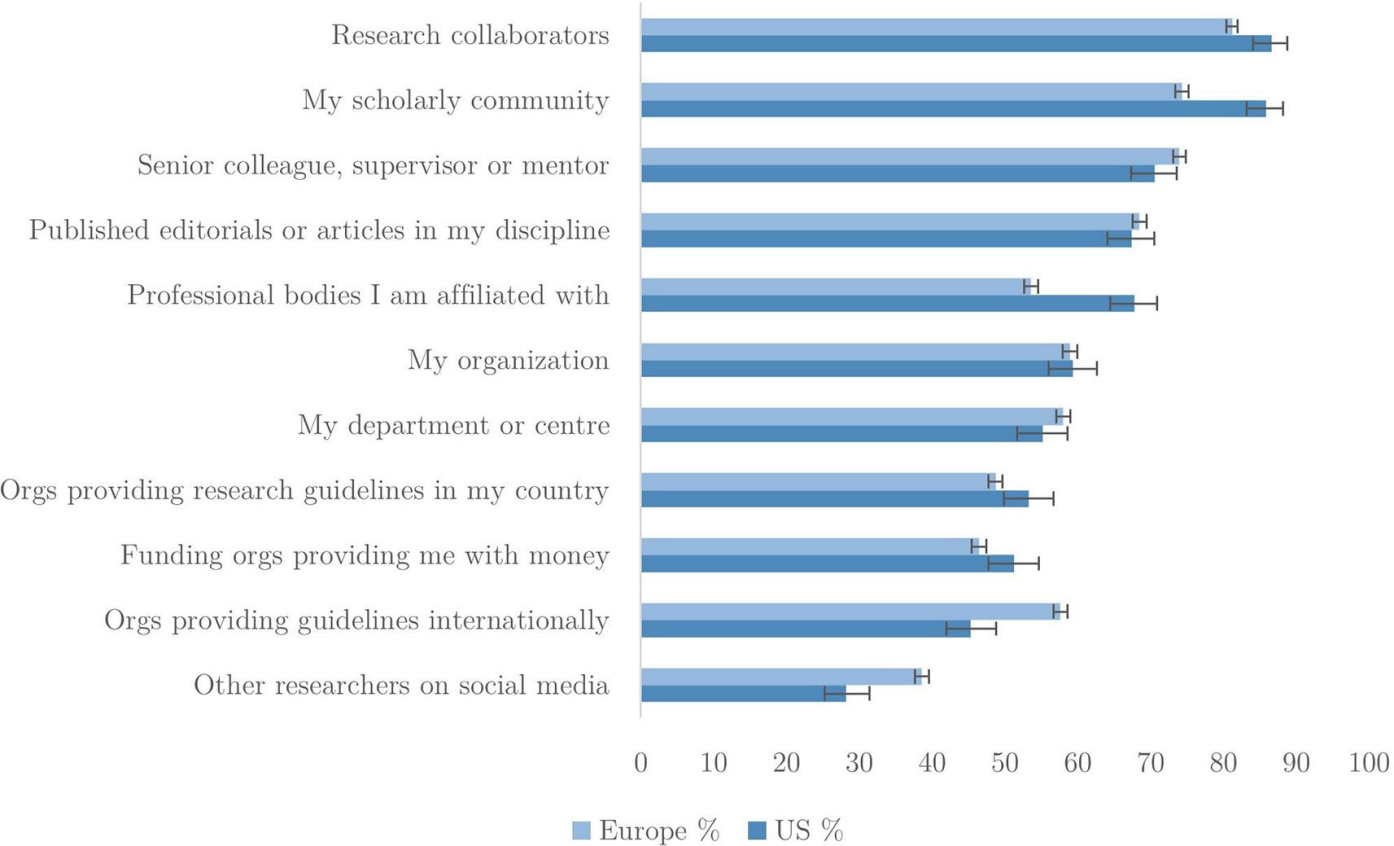
Percentage of researchers gaining knowledge from sources of information (n=35,930).

Where do researchers gain information about RI? Figure 4 shows the percentages who say they obtain ‘some’ or ‘a lot’ of knowledge from eleven sources. Both in the US and in Europe it is scholarly communities and research collaborators from whom the most knowledge is acquired. Perhaps unsurprisingly, there is a gradient in the amount of knowledge acquired from these more proximal sources (more opportunities, more contact) to more distant sources, such as funding bodies and academies committed to RI. Researchers’ organizations and departments are endorsed by around half of respondents as significant sources of information on RI. Thus, it appears that organizations have ready channels of information transmission to exploit.

### Enthusiasts and skeptics

Drilling deeper into the roles of organizations and researchers, and what the scope for action might be, respondents were asked the following. Firstly, whether they thought RI policies would help to improve the quality of their own research; secondly, whether they considered these policies to be genuinely intended for the purposes of improvement or simply for meeting bureaucratic requirements (‘box-ticking’), and finally how positive they would feel about receiving RI training. Responses to the three questions were combined into a categorical indicator to identify those who are more enthusiastic and likely to be more engaged and those who are generally skeptical towards organizational measures (see *Extended data*, Allum *et al.*, 2022).

**Figure 5.**
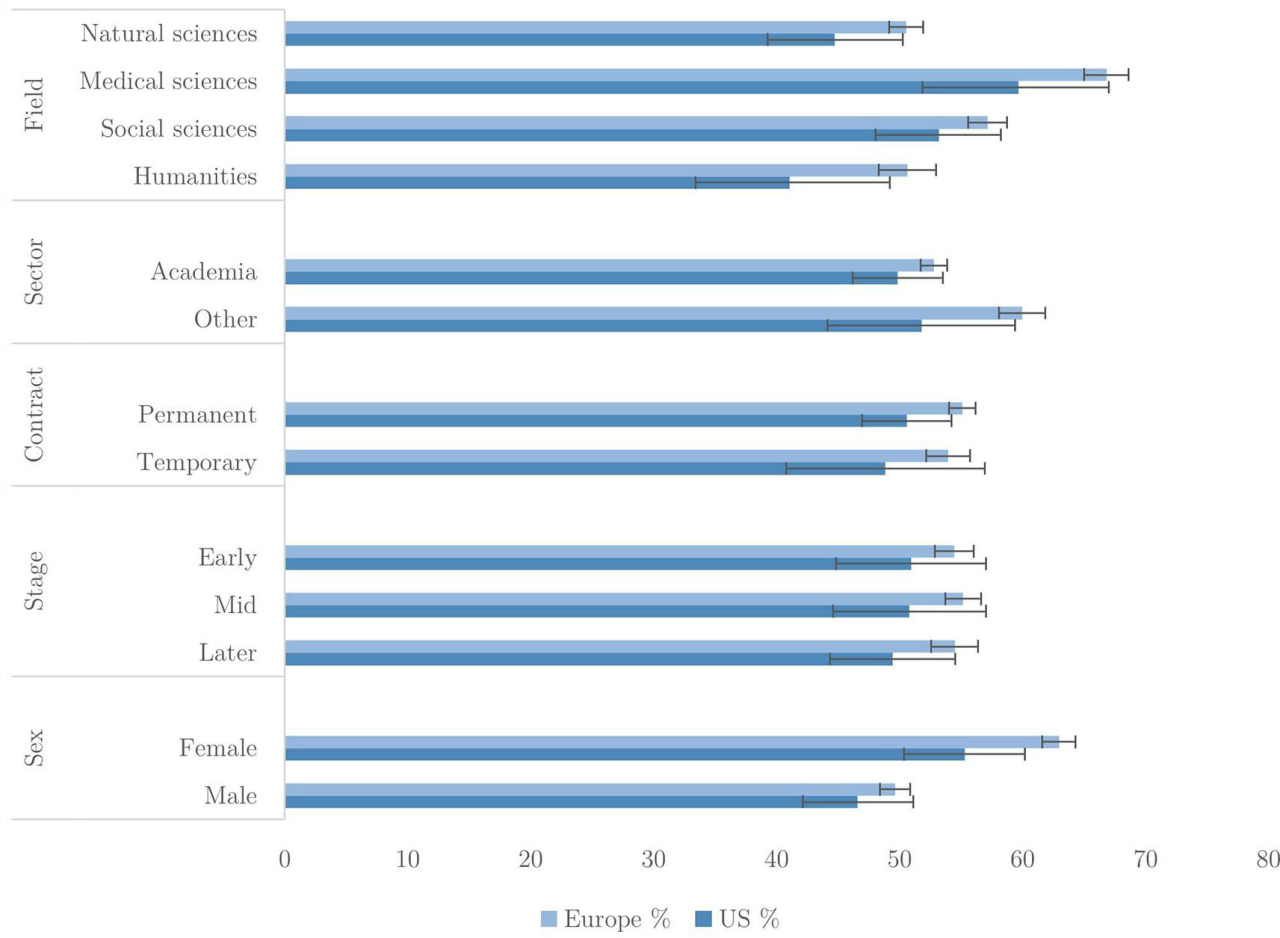
Percentage of researchers showing positivity towards RI measures (n=41,040).

**Figure 6.**
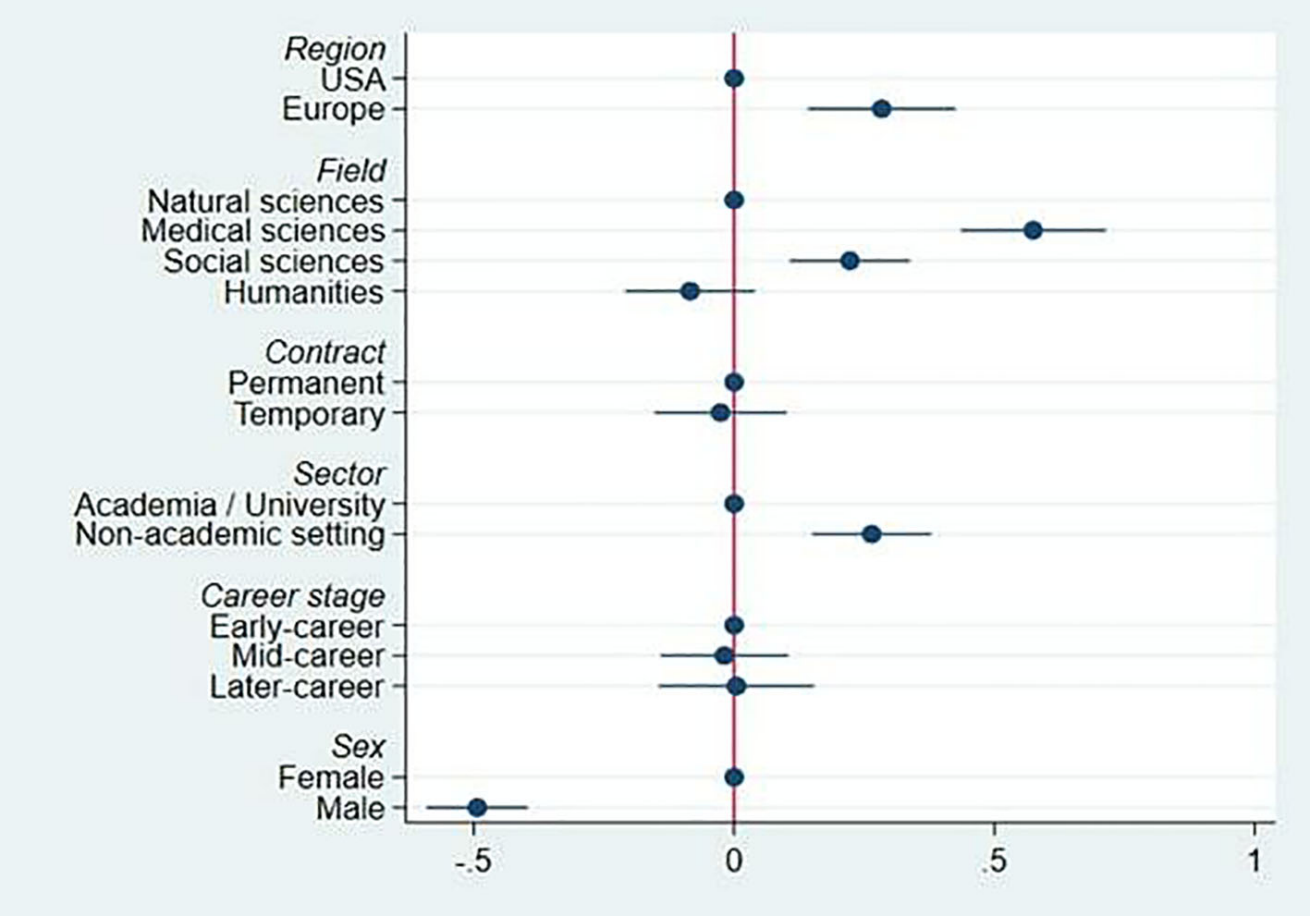
Regression of RI enthusiast score on country group, field, contract, sector, career stage and sex.

Figure 5 shows the percentages of enthusiasts for personal and organizational RI measures. Overall, researchers are significantly more enthusiastic, at 54%, than skeptical, at 30% (with neutrals at 16%). In absolute terms (chi square test), consistently across the demographic breakdowns, Europeans are more positive than American researchers, although the differences in most cases are not statistically significant. The highest levels of positivity are found amongst women, medical scientists and those working outside of the university sector. Figure 6 shows the results of a multivariate analysis showing the associations of the explanatory variables in Figure 5 considered as a whole. Region, field, sector and sex show robust differences, after adjusting for all other factors.

### Motivation

**Figure 7.**
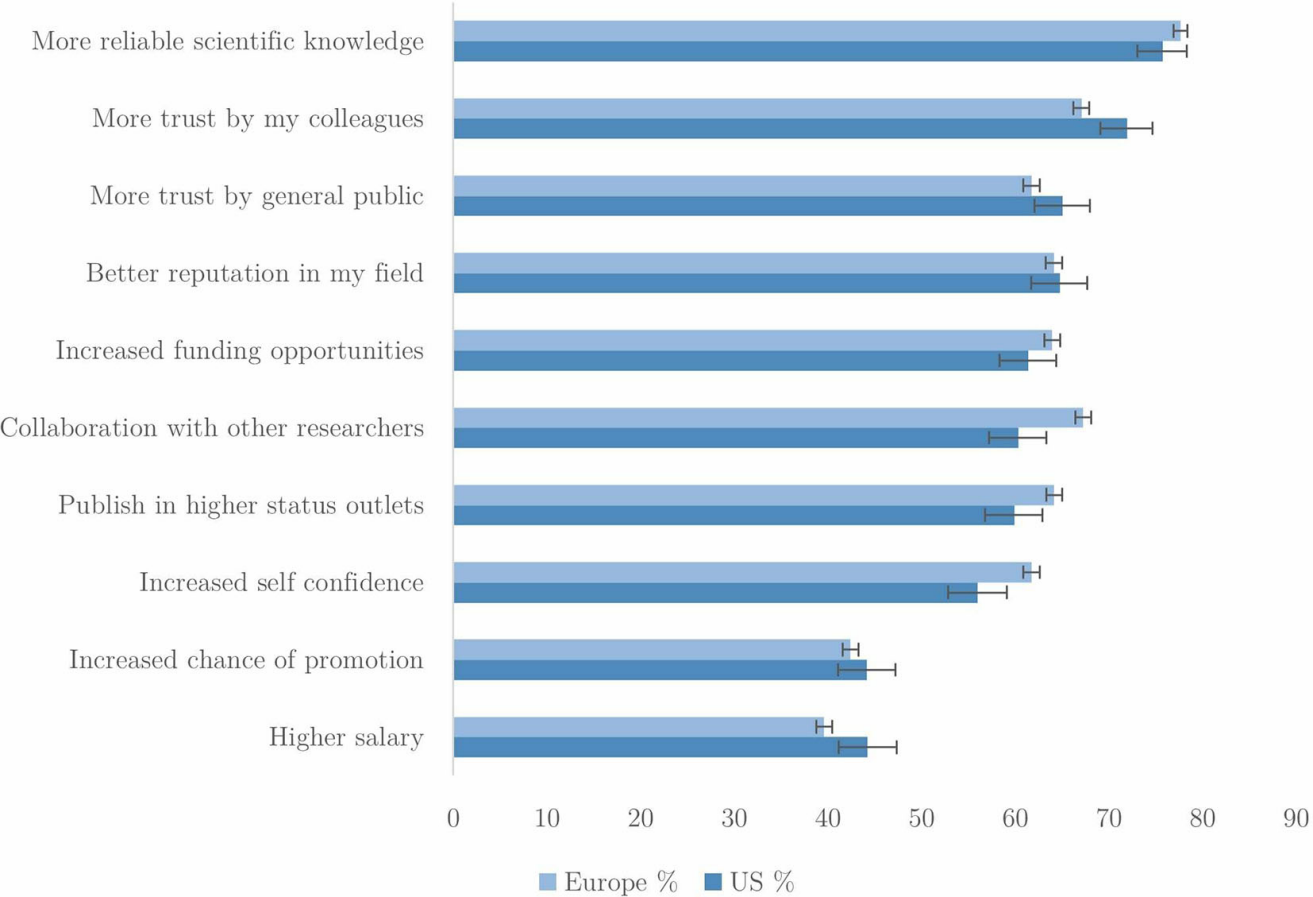
Percentage of respondents very or extremely motivated by benefits of following RI procedures (n=44,713).

For organizations to successfully implement appropriate procedures and protocols for ethical and responsible research, researchers must be motivated to engage with them. Based on previous qualitative research ( Sørensen *et al*., 2021; Sorensen and Ravn, 2020) we presented respondents with a list, shown in Figure 7, of possible motivations for complying with RI practices. We wanted to understand what researchers consider to be the benefits of engaging with these policies, and what kind of incentives could be important to them.

The top incentives for scientists in complying with RI procedures concern intrinsic scientific benefits, ‘truth’ and ‘trust’, specifically: *more reliable scientific knowledge; more trust from colleagues; more trust from the general public;* and *a better reputation in my field.* It is of note that the extrinsic benefits of promotion and salary prospects are reported to be the least motivating.

There are some differences between researchers based in US and Europe. In the US, the possibility of higher salary is more motivating than it is for Europeans. Europeans are more likely to be motivated by enhanced opportunities to collaborate with others and to gain more self-confidence.

### Additional support

**Figure 8.**
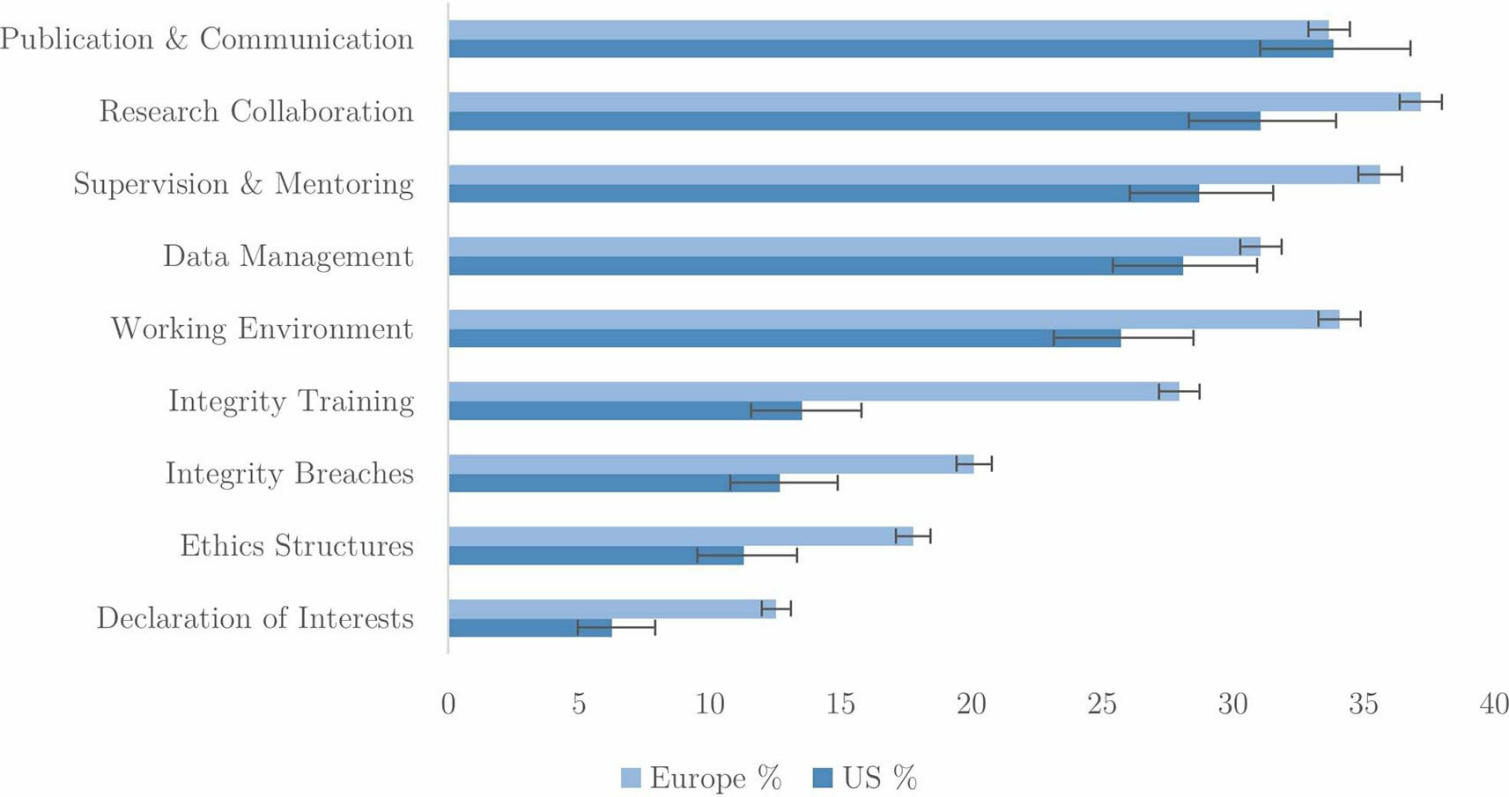
Percentage of researchers welcoming support in nine RI areas (n=47,906).

How do these overall attitudes and motivations translate into welcoming additional support in each of the nine RI areas? Figure 8 shows that between 20% and 35% of researchers in US and Europe would welcome further support on the majority of the nine RI topics, with European researchers showing greater demand than researchers in the US. Particularly striking is the fact that twice as many Europeans (28%) as Americans (14%) would welcome additional RI training. Additional support in cases of integrity breaches is also an issue where European researchers appear much more open than their American counterparts. This suggests that formal professional development programs in RI would be welcomed by many European researchers, although a majority in both regions remain unpersuaded.

## Discussion

In this paper we have examined researchers’ beliefs, values and behaviors, and what they think about their organization’s practices and performance. The results point to problems in workstyles, behaviours and organizational practices. Set against this, are positive signs of a readiness to adopt research and organizational practices which are believed to be conducive to enhancing responsible research conduct.

With the common experience of three decades of policy making on RI, four in five senior researchers in the US are confident about the integrity of their own research, while the same is true for only three in five in Europe, falling to two in five for Europe’s early career researchers. Confidence in their organizations’ effectiveness in supporting RI is expressed by less than half of Americans and less than a third of European researchers. Admissions of QRPs ranging from *superficial peer reviewing, selective reporting* and *researching without ethical approval*, are not uncommon and are more frequent in Europe than the US, most noticeably for *carrying out research without ethical approval* and *failing to disclose conflicts of interest.* In both cases, the rate of admission is twice as high for European researchers. In some instances, this may be indicative of sloppier practice in Europe, but it may also reflect variability in the formal requirements for ethical approval and declarations of interest in different national and institutional contexts.

Our estimates of the frequency of QRPs exceed those in the meta-analyses of Fanelli and more recently of Xie *et al.* ( Fanelli, 2009; Xie *et al*., 2021) but are more consistent with recent surveys carried out in the Netherlands and Norway ( Gopalakrishna *et al*., 2022; Kaiser *et al*., 2021).

Asked to assess whether their organization has high standards on nine RI topics, while agreement is higher in the US than Europe and among the medical science disciplines, overall, half of researchers say their organization does not have high standards.

There are, however, positive indications regarding the development of individual competences and organizational policies and practices. Researchers value the intrinsic benefits of RI, in terms of delivering greater research quality and trustworthiness far more than the extrinsic benefits of promotion and salary. Most researchers recognize that responsibility for RI is shared between them and their organization. In Europe a majority say they are willing to engage in RI training, consider that RI policies are well intentioned and should improve research quality. A non-trivial percentage of European and American researchers would welcome additional support across the nine RI topics.

What are the lessons of the IRIS survey for researchers and their organizations? While there is concern about the current state of health of integrity in research conduct and a recognition of its benefits, the findings from the US and Europe show that there are no magic bullets, no quick fixes. After three decades promoting ethics and integrity in research conduct, we find integrity deficits in both researchers’ behaviors and in how they judge their organization’s practices. This leads to a cornerstone in the framing of RI; it is the responsibility of multiple actors – researchers, their organizations, research funders, professional bodies and academies. A particular responsibility for research performing organizations (RPOs) is the provision of training in ethics and research conduct for all involved in the research cycle including students at all levels, researchers and administrators. The pro-active commitment of the NSF in the US shows that funding organizations have a vital role in setting the agenda on RI for research funding organizations (RFOs) and for monitoring compliance. Horizon Europe is in a unique position in Europe to make similar demands of RPOs and the researchers.

Changing organizational cultures and individual norms and standards of behavior towards greater RI will take time, commitment and resources. It is notable that researchers rely on many channels of communication about RI; their colleagues, departments, organizations, funding agencies and the academies, suggesting that the involvement of many different participants in the research system would be efficacious. Policies must be developed to reinforce best practice rather than being seen as an irrelevance to the real business of research. The SOPs4RI study has published guidelines for ‘Research integrity Promotion Plans’ which organizations may find useful in achieving a healthy research culture ( “Toolbox – SOPs4RI,” n.d.). The empirical foundations laid by the IRIS can provide clarity on how RI might be facilitated, and which actors should play a role, enabling comparisons across multiple dimensions, highlighting commonalities and divergences across groups, in the continued efforts towards heightened research integrity.

Finally, to our knowledge, this is the first dataset with which systematic comparison jointly between countries, regions and scientific fields has been possible. The IRIS data also allow for comparisons to be made between European countries, and there is much more insight that can be mined.

## Data Availability

Open Science Framework: International Survey on Research Integrity (IRIS), https://doi.org/10.17605/OSF.IO/XB9RK ( Allum *et al*., 2022). This project contains the following underlying data:
• Data file 1. (Full dataset in Stata and .csv formats) • Data file 2. (Stata replication code in .do format) Open Science Framework: International Survey on Research Integrity (IRIS), https://doi.org/10.17605/OSF.IO/XB9RK ( Allum *et al*., 2022). This project contains the following extended data:
• Data file 1. (Supplementary material (SM) and D6.2 Final report and recommendations) • Data file 3. (Survey protocol, questionnaire and ethics approval) Data are available under the terms of the Creative Commons Attribution 4.0 International Licence (CC-BY-4.0)

## Software availability

Stata 17 is available from StataCorp.
