# Supplementary material for: Researchers on research integrity: a survey of European and American researchers
Source: F1000Res. 2023 Feb 16;12:187. [Version 1] doi: 10.12688/f1000research.128733.1 (PMC10349267; doi:10.12688/f1000research.128733.1)
Supplement: Peer review history

# Peer review: Reviewer response for version 1

**DOI:** 10.5256/f1000research.141352.r182504

**Published:** 2023-07-18

**Authors:** Bibek Dahal

**Affiliations:** 1 Educational Research, University of Calgary, Calgary, Alberta, Canada

Very intense and insightful survey on research integrity! The methodological rigour is exceptional. The presentation of data along with interpretation helps to follow the survey results.  

 From the reader's viewpoint, I have realized the following rooms for improvement:  

1. A clear statement regarding the rationale of comparing a country (US) with a continent (EU) is required to understand the overall implication of this study.
2. It is better to unpack the concept of ‘responsible research practice’, ‘questionable research practice’, ‘detrimental research practice’ and ‘breaches of the principle/code of conduct’. This will help the readers to understand the meaning of these terms in this study's case.
3. It is better to explain with examples, how ‘questionable research practice’ is different than ‘research misconduct’, if these two terms were conceptualized differently during the survey in this study.
4. Write a conclusion explaining the implication of the study and suggestions for future research.    

Is the work clearly and accurately presented and does it cite the current literature?

Yes

If applicable, is the statistical analysis and its interpretation appropriate?

I cannot comment. A qualified statistician is required.

Are all the source data underlying the results available to ensure full reproducibility?

Yes

Is the study design appropriate and is the work technically sound?

Yes

Are the conclusions drawn adequately supported by the results?

Partly

Are sufficient details of methods and analysis provided to allow replication by others?

Yes

Reviewer Expertise:

Academic Integrity, Research Integrity, Research Ethics, Knowledge Equity in Higher Education

I confirm that I have read this submission and believe that I have an appropriate level of expertise to confirm that it is of an acceptable scientific standard.

# Peer review: Reviewer response for version 1

**DOI:** 10.5256/f1000research.141352.r182505

**Published:** 2023-07-14

**Authors:** Simon Kolstoe

**Affiliations:** 1 School of Health & Care Professions, University of Portsmouth, Portsmouth, England, UK

I enjoyed reading this study, and especially the fact that it focussed on both European and American researchers. Of course this misses out the rest of the world, but you do have to start somewhere! It was also reassuring that they managed such a large sample size.

 Assessments as to the reliability of research are clearly essential to the overall value of research. In recent years this assessment (some may call it a concern) has been given various names, one of which is research integrity. In once sense integrity can be considered as quite specifically focused on the data itself, however a more useful way to use the phrase is to consider the overall behaviour of researchers, wherein a researcher acting with high levels of integrity will subsequently produce reliable data (data integrity) but more widely will also contribute to the creation and maintenance of a healthy research culture. The opposite of acting with integrity is thus engaging in questionable research practices (QRPs). As a consequence trying to determine levels and trends in QRPs is essential if trying to assess the current levels of integrity within the research community, and identify areas where more work is required.

 This paper adds to the literature by providing an up to date survey of QRPs alongside gathering information on what researchers think about research integrity practices/initiatives. The large sample size, sophisticated sampling strategy, and comparison between the US and EU provide a level of reassurance that the data presented here is more than just an interesting anecdote.

 Thus said the research community is wide with a large variety of roles, academic practices, and career pathways. As a consequence any large survey is bound to miss subtleties no matter how careful the analysis, so I do not entirely agree that a survey like this is truly representative (as claimed by the authors). But again, the authors have done a good job of trying to break down the data into relevant subgroups, that will add constructively to wider discussions.

 As an aside, I was astounded that the pilot testing involved 5000 people, surely about 50 or so would have sufficed? Also, as I am not a statistician, I cannot really pass comment on the details of the sample selection and analysis, but the technical description does at least provide a level of existential reassurance that this was carefully thought through!

 Are the results novel or surprising - possibly not for those familiar with this area, but what they are is relatively systematic and, as mentioned above, more than the anecdotal figures that we normally use to talk about these things. So from this perspective this paper (and data therein) is definitely a valuable contribution, and one that I have already referenced in a plenary conference presentation. Indeed I also note that the newly established UK Committee on Research Integrity (UKCORI) also referenced this data in their first annual report, again providing evidence of its value.

 There are, of course, weaknesses, or at least points that can be objected to by a reviewer. For instance I would disagree with the statement that " *[QRPs]... fall short of outright misconduct but represent transgressions of best practice*". Increasingly QRPs are seen as a spectrum with innocent errors on the minor end, and misconduct on the major end ^1^. Likewise, the eight QRPs shown in Figure 1 seem to be selected somewhat arbitrarily and are perhaps not the ones I would have chosen. Indeed if, as I contend above, QRPs represent a spectrum with misconduct at one end, I would have wanted to ask about ignoring datapoints, fabrication, and covering up inadequate analyses.

 I also wonder how many of the differences between the US and EU researchers come down to definitions that are not quite the same on both sides of the Atlantic. US culture and world view is increasingly divergent from Europeans, and while we try to communicate in the same language, the baggage that comes with certain statements or ideas can be quite different. Thus said, it is fascinating that US researchers seem to have more confidence in their institutions handling of RI, despite Americans generally being considered as being less trusting of institutions and governance structures. However, conversely, the US researchers are also less enthusiastic about RI measures in general. A qualitative analysis of such views is about the only way these issues could be further explored (and given the size of the project that this work comes from, I will look forward to reading this analysis in due course!).

 Finally I was bemused by the statement: " *four in five senior researchers in the US are confident about the integrity of their own research, while the same is true for only three in five in Europe, falling to two in five for Europe’s early career researchers." *I wonder whether, far from being an indictment on the quality of European research (which was perhaps implied in the discussion), this may instead be an indication that European researchers are more realistic about the nature of research and the serious challenges that need to be addressed?

Is the work clearly and accurately presented and does it cite the current literature?

Yes

If applicable, is the statistical analysis and its interpretation appropriate?

I cannot comment. A qualified statistician is required.

Are all the source data underlying the results available to ensure full reproducibility?

Partly

Is the study design appropriate and is the work technically sound?

Yes

Are the conclusions drawn adequately supported by the results?

Yes

Are sufficient details of methods and analysis provided to allow replication by others?

Yes

Reviewer Expertise:

Research Ethics, Integrity and Governance

I confirm that I have read this submission and believe that I have an appropriate level of expertise to confirm that it is of an acceptable scientific standard.

## References

[1] : Defining the Spectrum of Questionable Research Practices (QRPs).2023; 10.37672/UKRIO.2023.02.QRPs 10.37672/UKRIO.2023.02.QRPs

# Peer review: Reviewer response for version 1

**DOI:** 10.5256/f1000research.141352.r163833

**Published:** 2023-02-22

**Authors:** Dorothy Vera Margaret Bishop

**Affiliations:** 1 Department of Experimental Psychology, University of Oxford, Oxford, UK

This is a very nice piece of work that provides important information on research integrity.  It is impressive because not only is there a large sample size, but the sampling strategy was far more sophisticated than in previous surveys of this kind.  A great deal of thought has gone into the survey design. A complex data set is clearly presented with appropriate graphics, and the full dataset is available for others to peruse.

 I have only minor suggestions for improvement.

1. I was initially unclear whether author position was taken into account in either selecting respondents or in analysis. I think it wasn’t, which is fine, but it may be worth explicitly stating that.
2. Similarly, I assume no incentives were used to encourage people to respond?
3. I have a particular interest in how whistleblowers are treated by institutions, and so I looked at the reference at the end of para 2 of the introduction. It was not clear that this applied to academics: It seemed to come from the Department of Homeland Security. Some clarification would help here.
4. Use of commas as numeric separator for thousands is inconsistent in text and in figures/tables.
5. In line 9 of paragraph p 5 Analysis/statistical methods, the letter f is twice replaced by another font – I noticed this as it did not print on my printer.
6. Tables 2  and 6 – my preference would be for these to be reformatted so the top level is US vs Eur, and the next level is confidence level. I found it quite difficult to scan across the table to appreciate the differences, especially with on-screen viewing.  This is particularly true for Table 6, where the interest is in how many scores are on the diagonal.  I don’t see this as essential: all the information is there, but maybe change if others agree it could be more clearly laid out.
7. I was initially confused in Figure 1 by “Not conducting a thorough peer review”, as I had thought the questions pertained to one’s own research, so I wondered if it meant the researcher had not adequately evaluated their own protocol before doing the study – or perhaps had published work in non-peer-reviewed outlets. Subsequently, I realised this was referring to the case when researchers peer review the work of others. I think minor rewording could clarify this.
8. P 14 , on incentives. “It is of note that the extrinsic benefits of promotion and salary prospects are reported to be the least motivating”.  I think this might need unpicking a bit. A major problem in my experience is that researchers tend to believe that in order to advance their careers it helps to adopt QRPs!  So my suspicion is that researchers are certainly motivated by promotion and salary prospects, but they just don’t believe that the way to achieve these is by working with integrity – indeed quite the opposite is often assumed. I assume you don’t have data to test that interpretation, but it may be worth flagging up.

Is the work clearly and accurately presented and does it cite the current literature?

Yes

If applicable, is the statistical analysis and its interpretation appropriate?

Yes

Are all the source data underlying the results available to ensure full reproducibility?

Yes

Is the study design appropriate and is the work technically sound?

Yes

Are the conclusions drawn adequately supported by the results?

Yes

Are sufficient details of methods and analysis provided to allow replication by others?

Yes

Reviewer Expertise:

Neuropsychology, Child Development, Research Integrity

I confirm that I have read this submission and believe that I have an appropriate level of expertise to confirm that it is of an acceptable scientific standard.
